# Nutritional, Functional, and Microbial Quality of Wheat Biscuits Enriched With Malted Pearl Millet and Orange Peel Flours

**DOI:** 10.1002/fsn3.4562

**Published:** 2024-11-12

**Authors:** Shonisani Eugenia Ramashia, Matimu Delicate Ntsanwisi, Oluwatoyin Oladayo Onipe, Mpho Edward Mashau, Gbeminiyi Olamiti

**Affiliations:** ^1^ Department of Food Science and Technology, Faculty of Science, Engineering, and Agriculture University of Venda Thohoyandou South Africa

**Keywords:** biscuit antioxidant capacity, functional properties, malted pearl millet, microbial quality, orange peel flour

## Abstract

In this study, composite biscuits were produced by combining wheat flour (WF) with different proportions of malted pearl millet (MPM) flour (8%, 16%, 24%, and 32%) and orange peel (OP) flour (2%, 4%, 6%, and 8%), using 100% WF as a control. The investigation covered the functional properties, viscosity, and thermal properties of the flours, along with the proximate composition, antioxidant, physical properties, color attributes, and microbial quality of the composite biscuits. As MPM and OP flour (OPF) contents increased, water absorption capacity, dispersibility, and foaming power increased, while the viscosities of both hot and cold pastes decreased. The thermal properties of the composite flours, including onset, peak, and final temperatures (ranging between 74.19°C and 100.76°C), showed an upward trend with increasing proportions of MPM and orange peel flour (OPF). There was an increase in moisture content (3.43%–4.93%), ash (4.50%–5.59%), crude protein (11.70%–13.41%), and crude fiber (11.44%–16.24%) of biscuits with the incorporation of MPM and OPF. Similarly, the diameter (4.12–4.60 mm), thickness (9.00–10.00 mm), and hardness (7.53–8.75 N) of the biscuits were increased. Antioxidant properties were evident, with an increased total phenolic content (1.40–3.56 mg GAE/100 g), total flavonoid content (2.91–6.79 mg QUE/100 g), vitamin C (0.79–1.01 mg/g), and ferric reducing antioxidant power (1.78–8.64 mg GAE/g). Conversely, color attributes—L* (31.90), a* (10.82), b* (19.59), hue angle (30.42), and chroma (53.66)—were found to decrease with higher levels of MPM and OPF. Microbial quality showed decreased total counts, coliforms, yeasts, and mold in biscuits containing MPM and OPF. Overall, the inclusion of MPM and OPF enhanced the nutritional quality of the biscuits and could reduce reliance on imported wheat.

## Graphical abstract

Nutritional, functional, and microbial quality of wheat biscuits enriched with malted pearl millet (MPM) and orange peel (OP) flours were determined. The increase in the percentage of substitution of MPM and OP flours (OPFs) significantly increased the ash, protein, crude fiber contents, and bioactive compounds of the formulated biscuits. Furthermore, the diameter, thickness, and hardness of the biscuits were increased. The incorporation of MPM and OPFs decreased the microbial quality of the formulated biscuits.

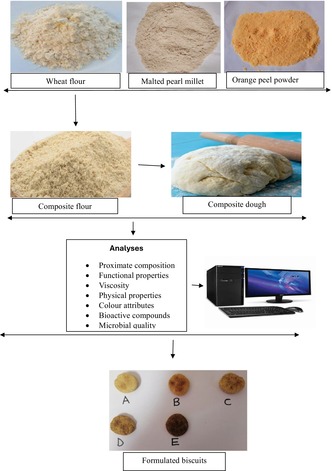

## 1 Introduction

Biscuits, known as cookies in certain regions, are delectable baked goods primarily crafted from wheat flour (WF). They are typically characterized by their diminutive, flat, and circular shapes. Biscuits have a wide range of taste, texture, and ingredients across diverse cultures and localities, which contributes to their extensive global appeal (Mishra and Mishra 2024). Conventionally, biscuits are composed of refined flour, oil, sugar, and other components that enhance their distinct flavors, resulting in their widespread popularity and convenience (Gutierrez‐Barrutia et al. 2023). Nonetheless, it is crucial to acknowledge that most ready‐to‐eat foods, including biscuits and quick snacks, lack sufficient nutritional value (George and Ravishankar 2022). Despite providing immediate satisfaction, biscuits are often high in calorie content, deficient in dietary fiber, and lacking vital minerals. To cultivate healthier eating habits, it becomes imperative to evaluate and enhance the nutritional composition of the bakery products such as biscuits (Arepally et al. 2020).

Pearl millet (*Pennisetum glaucum*) is a versatile cereal crop widely cultivated in African and Asian countries. It serves diverse purposes, such as food, feed, and forage. This resilient crop exhibits exceptional tolerance to drought and high temperatures, making it well‐suited for cultivation in regions where other cereal crops like wheat, maize, and other cereals face challenges in thriving. Despite Africa's significant contribution to pearl millet production, its utilization remains underexploited (Shrestha et al. 2023). Compared to regular wheat‐based meals, incorporating pearl millet into one's diet offers numerous health benefits. It is unable to trigger celiac disease and other forms of gluten sensitivity. As the demand for gluten‐free products continues to rise, the nutritional advantages of pearl millet have garnered attention and popularity. Pearl millet stands as an excellent choice for enhancing food diversity due to its high content of fiber, minerals, proteins, and antioxidants. These are comparable to or even higher than those found in other staple grains like rice and maize in their natural form (Taylor 2016).

Orange peel (OP), the outer layer of the orange fruit skin (*Citrus sinensis*), is versatile and allows for its widespread use in cooking. It enhances the flavor and aroma of various dishes and desserts. OP contains essential oils, antioxidants, and dietary fiber, contributing to its potential health benefits. Extensive research has identified several compounds in OP, including flavonoids, carotenoids, and vitamin C, giving it antioxidant properties. These antioxidants are crucial in neutralizing harmful free radicals and protecting against oxidative stress (Fontana 2021; Matsuzaki et al. 2022; Rafiq et al. 2018). Furthermore, OP exhibits potential antimicrobial, anti‐inflammatory, and anticancer properties. Specific compounds found in OP, such as limonene and hesperidin, have been extensively researched for their antimicrobial effects and have shown promise in combating various pathogens as well as their anti‐inflammatory properties (Hernández et al. 2021). Moreover, studies have indicated that OP's soluble fiber, pectin, may contribute to the reduction of cholesterol levels in the body (Nazir et al. 2022). This discovery further enhances understanding of the health‐promoting characteristics of OP and emphasizes its value as a beneficial addition to the diet. Additionally, OP represents a viable and nutritious source for fortifying food products. The incorporation of OP into composite flours presents exciting possibilities for enhancing the nutritional composition of different food items and can play a vital role in offering consumers healthier and more nourishing food choices (Rafiq et al. 2018).

Wheat flour serves as a fundamental component in the majority of biscuit recipes and is widely recognized for its exceptional ability to create dough. As a staple crop consumed on a global scale, wheat is an indispensable commodity due to its gluten‐rich proteins that provide essential extensibility and elasticity, which are crucial for the production of various bakery products and pasta (Girard and Awika 2020).

The incorporation of OP in bakery products has been documented in different studies. Rani, Sangwan, and Malik (2020) reported that the contents of total insoluble and soluble dietary fibers, total phenolic content (TPC), and radical scavenging activity were significantly higher in OP flour (OPF)‐incorporated biscuits than in control biscuit. On the other hand, Gasparre et al. (2024) reported that OP enhanced nutritional profiles, including higher ash and dietary fiber content of gluten‐free flatbread. However, despite the well‐documented nutritional benefits of wheat, pearl millet, and OP, there is a lack of research on the combined effects of these ingredients on biscuit production. Therefore, the objective of this study was to investigate the nutritional, functional, and microbial quality of wheat biscuits enriched with malted pearl millet (MPM) flour and OP powder. By exploring the potential synergies among these ingredients, this research provides valuable insights into creating innovative and nutritious biscuit formulations. These scholarly efforts are crucial for diversifying food options and promoting healthier dietary choices worldwide.

## 2 Material and Methods

### 2.1 Sample Collection

Wheat flour, margarine, baking powder, sugar, and eggs were obtained from local supermarkets in Thohoyandou, Limpopo Province, South Africa. Additionally, pearl millet grains and oranges were purchased from supermarkets in the same region. The equipment utilized included a hammer mill, stainless steel flour sieve (0.5 mm), digital weighing scale, moisture analyzer, measuring cylinder, hot air oven, and differential scanning calorimeter. All solvents and reagents employed in this investigation were of analytical grade and were procured from Merck, South Africa.

### 2.2 Sample Preparation

#### 2.2.1 Malting of the Pearl Millet

**FIGURE 1:**
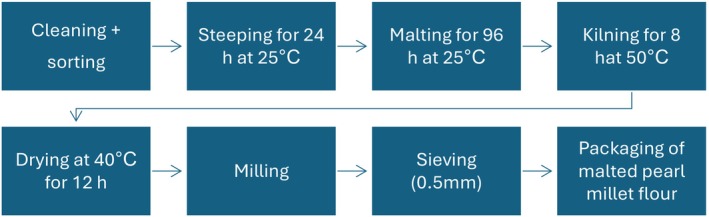
Flowchart for MPM flour.

Pearl millet grains (100 g) were immersed in water at 25°C for 24 h. Following the soaking process, the grains were spread out onto sanitary paper towels and maintained in a moist state through the periodic sprinkling of water at intervals of 24 h. Subsequently, the soaked grains were allowed to germinate for 96 h at a temperature of 25°C, after which the sprouted grains were subjected to kilning for 8 h at a 50°C. Postkilning, the grains were carefully dried and finely ground into flour using a Hammer mill (Hammer‐type mill 120 Perton). The resulting flour was then stored in polyethylene zip bags at a temperature of 20°C until further analysis (Olamiti et al. 2020) (Figure 1).

#### 2.2.2 Preparation of OPF

Fully ripe oranges were thoroughly cleaned and peeled, and the pulp was separated, leaving only the OP sliced into pieces < 2 mm thick. Chemical debittering of the OPs was conducted using the method proposed by Wang et al. (2021) with minor modifications. Debittering was done through a sequential alkali treatment, followed by acidic treatment. The debittering procedure involved washing the pulverized peels in a 5% alkali solution (NaOH solution) for varying durations of 60 min at 40°C in a water bath (Sanco 220/230 V Waterbath). During the alkali treatment, pulverized OPs were mixed with NaOH at 1:40 (w/v). Subsequently, the alkali‐treated peels were neutralized with a 1% acid solution (citric acid solution) in a water bath for 120 min at 40°C. The peel samples were then rinsed with distilled water, subjected to chemical analysis, and dried in an oven at 30°C for 48 h. Once thoroughly dried, the peels were finely ground using a Hammer mill (Hammer‐type mill 120 Perton) and passed through a 60‐mesh screen to obtain OPF. To maintain the quality of the flour, all samples were securely sealed in polyethylene bags and stored in cold storage at 5°C for subsequent analysis.

#### 2.2.3 Formulation of Composite Flour Biscuits

For each composite biscuit, 250 g of flour blend, selected from formulations A, B, C, and D (Table 1), was utilized. The ingredients included 125 g of margarine, 50 g of sugar, 1 large egg, and 3.75 g of baking powder, following the baking procedure outlined by Ramashia, Mamadisa, and Mashau (2021).

**TABLE 1:** Composite flour formulations.

Flour blend	WF (%)	MPM flour (%)	OPF (%)
A (control)	100	—	—
B	90	8	2
C	80	16	4
D	70	24	6
E	60	32	8

*Note:* WF incorporated with MPM and OPF biscuits, A = 100% WF; B = 90:8:2 (WF:MPM:OPF); C = 80:16:4 (WF:MPM:OPF), D = 70:24:6 (WF:MPM:OPF) and E = 60:32:8 (WF:MPM:OPF) (Ramashia, Mamadisa, and Mashau 2021).

Abbreviations: MPM, malted pearl millet; OPF, orange powder flour; WF, wheat flour.

The biscuit‐making process commenced by combining flour, sugar, baking powder, and margarine blends. Eggs were then added to create a cohesive dough. The dough was subsequently kneaded flat until it reached a smooth consistency. It was then rolled out into sheets using a rolling pin and cut into various shapes using plastic cutters. These cut‐out shapes were carefully placed onto baking trays in a preheated oven set at 180°C. The baking time ranged from 10 to 15 min, ensuring a delightful golden‐brown color was achieved. After baking, the biscuits were allowed to cool at room temperature (25°C) before being stored in low‐density zipper bags for subsequent analysis. Control sample, consisting of biscuits made entirely from WF (100%), was also prepared.

### 2.3 Functional Properties of the Composite Flour

#### 2.3.1 Bulk Density (BD) of Flour

Five grams of flour was accurately measured and placed into a 100 mL measuring cylinder. The cylinder was gently tapped on a laboratory table several times until a consistent volume was reached, following the methodology outlined by Ojediran, Adamu, and Jim‐George (2021). Afterward, the BD was calculated by dividing the weight of the flour (in grams) by its corresponding volume (in cm^3^).

(1) $$\text{Bulk density}=\frac{\text{mass}\;\text{of flour}\;\left(g\right)}{\text{volume of flour}\;\left({\mathrm{cm}}^{3}\right)}$$

#### 2.3.2 Swelling Capacity (SC) of Flour

One gram of flour was accurately measured and mixed with 10 mL of distilled water in a centrifuge tube. The mixture was then heated to 80°C for 30 min while being vigorously agitated to ensure proper dispersion. After that, the suspension was centrifuged for 15 min at 4000 rpm using a Biosan LMC‐3000 Laboratory Centrifuge (Model LV‐1067, Riga, Latvia). The resulting paste was evaluated by carefully decanting and weighing the supernatant, following the methodology described by Zhou et al. (2021).

The SC was subsequently calculated below.

(2) $$\text{Swelling capacity}=\frac{\text{mass of the paste}}{\text{weight of}\;\mathrm{dry}\;\text{flour}}$$

#### 2.3.3 Water Holding Capacity and Oil Absorption Capacity (OAC)

The water absorption capacity (WAC) and OAC of the flour were determined following the modified technique of Aidoo et al. (2022). For WAC, 1 g of flour was weighed and placed into 15 mL centrifuge tubes. Subsequently, 10 mL of distilled water was added to each sample and stirred for 2 min to guarantee thorough mixing. After allowing the samples to stand at room temperature for 30 min, they were centrifuged at 3000 rpm for 20 min. The volume of the decanted supernatant fluid was measured to determine the WAC in grams of water per gram of flour. Similarly, for OAC, 1 g of flour was added to a 10 mL of sunflower oil in a 15 mL centrifuge tube. After resting the sample for 30 min and centrifuged for 20 min at centrifugal force, the volume of the decanted supernatant fluid was measured to determine the OAC in milliliters of oil held per gram of flour.

#### 2.3.4 Foaming Capacity of Flour

In a 50 mL measuring cylinder, approximately 1 g of the flour sample was carefully added to 25 mL of distilled water. After 30 s, the suspension was thoroughly mixed and vigorously shaken to generate foam. The resulting total volume was then recorded, following the methodology described by Okpala and Mamah (2001). The foaming capacity was subsequently determined as the percentage increase in volume observed after the initial 30‐s period.

(3) $$\begin{array}{c} \text{Foaming capacity}\;\left(\%\right)\\ \;=\frac{\text{volume after whipping}-\text{volume before whipping}}{\text{volume after whipping}}\times 100 \end{array}$$

#### 2.3.5 Dispersibility of Flour

Ten grams of flour was accurately weighed and introduced into a 100‐ml measuring cylinder, subsequently followed by the addition of distilled water up to the 50‐ml mark. The mixture was vigorously stirred and left undisturbed for 3 h. Following this resting period, the volume occupied by the settled particles was measured, and this value was deducted from 100. The obtained result was then reported as the percentage dispersibility following the methodology elucidated by Jaddu, Pradhan, and Dwivedi (2022).

### 2.4 Viscosity (Hot and Cold Pastes) of Flour

Ten grams of flour was combined with 90 mL of distilled water and allowed to hydrate for 30 min at a temperature of 30°C. The mixture was regularly stirred to ensure complete dispersion, following the method outlined by Ramashia (2018). Afterward, the viscosity of the resulting slurry was determined by using a Brookfield viscometer (Model RV, Brookfield Engineering, Inc., USA), with the Q3 spindle rotating at a speed of 100 rpm. The viscosity of the cold paste was measured and recorded in centipoise (cP). The slurry was then heated in a water bath, boiled for 20 min at a temperature of 95°C, and then cooled back to 30°C, following the established methodology. The viscosity of the hot paste was then measured using the same viscometer setup and recorded in cP.

### 2.5 Thermal Analysis of Composite Flour

A differential scanning calorimeter (DSC 4000, Perkin‐Elmer, Shelton, CT, USA) was used to determine the gelatinization temperatures of flour samples. The equipment was calibrated with an indium. Four milligrams of flour was accurately weighed into aluminum pans to create a starch‐water suspension. Distilled water was added using a micro‐syringe. The pan was then sealed tightly and allowed to sit for 1 h at a controlled temperature of 20°C–25°C, with a relative humidity of 35%–50%, depending on the laboratory's environmental conditions. To act as a control, an empty pan was used and heated at 10°C/min from 20°C to 125°C. Thermal analyses were conducted to determine the start temperature (To), peak temperature (Tp), end temperature (Te), and gelatinization enthalpy (ΔH). The experiments were carried out using Pyris software (Perkin‐Elmer, Shelton, CT, USA), and these parameters were automatically determined (Olamiti et al. 2020).

### 2.6 Proximate Composition of Composite Biscuits

The proximate composition of the ground biscuits was analyzed using standard methods outlined by AOAC (2016). This included measuring moisture (method 945.32), ash (method 923.03), fat (method 920.39), crude protein (method 978.02), and fiber (method 991.43) separately. The carbohydrate content was determined using the difference method, as specified by Ganogpichayagrai and Suksaard (2020). To calculate the final energy conversion report, the energy content of the biscuit samples was determined using the conversion formula provided by García‐Martínez et al. (2020).

### 2.7 Total Polyphenol Content and Antioxidant Activity of Composite Biscuits

#### 2.7.1 Preparation of Phenolic Extracts

A total of 2 g of flour was mixed thoroughly with 16 mL of methanol containing 1% HCl. The mixture was then left to stand for 24 h at 24°C. After the extraction period, the methanol extracts were subjected to centrifugation using a Biosan LMC‐3000 Laboratory Centrifuge (Model LV‐1067, Riga, Latvia) at 4000 rpm for 15 min, following the method described by Alara, Abdurahman, and Ukaegbu (2021). The resulting supernatants were carefully collected, combined, and stored at 4°C for future use.

#### 2.7.2 Total Phenolic Content

A 0.5 mL of the extract was mixed with 5 mL of a Folin–Ciocalteu reagent and neutralized with 4 mL of a saturated sodium carbonate solution (75 g/L). The mixture was then stored at room temperature for 2 h to allow for proper reaction, following the methodology outlined by AlFaris et al. (2021). The TPC was quantified as gallic acid equivalents (mg GAE/100 g) using a spectrophotometer at a wavelength of 765 nm. The measurements were performed using a WPA Biowave II UV/Vis Spectrophotometer manufactured by Fisher Scientific Ltd., UK. The standard curve was created using gallic acid; results were expressed as milligrams of gallic acid equivalents per gram of dry weight.

#### 2.7.3 Total Flavonoid Content

A total of 0.5 mL of diluted extract was transferred into 15 mL polypropylene conical tubes. Each tube contained 2 mL of distilled water and 0.15 mL of 5% NaNO_2_ solution. After a 5‐min incubation period, 0.15 mL of 10% AlCl_3_‐H_2_O solution was added. The mixture was allowed to stand for an additional 5 min. Subsequently, 1 mL of 1 M NaOH was added, and the solution was thoroughly mixed. The solution was then left to stand for 15 min. The absorbance of the resulting solution was measured at a wavelength of 415 nm. Total flavonoid content was determined by constructing a standard Quercetin curve and expressing the results as Quercetin equivalents (mg QUE/100 g), following the methodology described by Zawawi et al. (2021).

#### 2.7.4 Determination of Ascorbic Acid (Vitamin C)

A 2.5 g of each composite biscuit sample was homogenized in 250 mL of distilled water for 5 min using vortexing, followed by incubation in a water bath at room temperature (30°C) for 10 min. Subsequently, the mixture was centrifuged at 2000 rpm for 10 min at room temperature, and the resulting supernatant was used for vitamin C determination. Vitamin C content was determined by employing the Oxidation–Reduction Titration method. A 50 mL burette was prepared on a ring stand, filled with 30:300 iodine solution. Meanwhile, 20 mL of the extract solution was poured into a 50 mL flask, and approximately 10 drops of starch indicator solution were added and stirred into the flask. The iodine solution was added drop by drop into the flask until the ascorbic acid titration was complete. The completion of titration was indicated by the appearance of a distinctive blue/black color in the solution, signifying the reaction of iodine with starch after all the vitamin C had been oxidized. The volume of iodine required to titrate the extract was measured, and the provided equation was used to determine the vitamin C content, as described by Savych and Basaraba (2021).

(4) $$\begin{array}{c} \text{Ascorbic acid}=\frac{20\;\mathrm{mg}\;\text{Vitamin}\;C}{\mathrm{mL}\;\text{iodine to titrate vitamin}\;C\;\text{in standard}}\\ \;=\frac{\mathrm{mg}\;\text{of Vitamin}\;C\;\text{in flour}}{\mathrm{mL}\;\text{iodine to titrate vitamin}\;C\;\text{in flour}} \end{array}$$

#### 2.7.5 Ferric Reducing Antioxidant Power

The ferric‐reducing antioxidant power was determined using the method described by Olamiti et al. (2020). First, 100 mL of the extract was added to a test tube, and the volume was adjusted to 1 mL using methanol. Then, 2.5 mL of 0.2 M phosphate buffer (pH 6.6) and 2.5 mL of 1% potassium ferricyanide were added to the tube, which was then vortexed. The mixture was incubated in a water bath at 50°C for 20 min. After incubation, 2.5 mL of 10% (w/v) trichloroacetic acid was added, and the resulting mixture was centrifuged for 20 min at 5000 rpm. The absorbance of the supernatant was measured at 700 nm using a spectrophotometer. To measure, 2.5 mL of the supernatant was combined with 2.5 mL of distilled water and 0.5 mL of 0.1% (w/v) ferric chloride in a test tube. Higher absorbance values indicate greater reducing power.

### 2.8 Physical Properties of the Composite Biscuits

#### 2.8.1 Diameter

About three biscuits were arranged in a line to measure their diameter (D). The overall diameter of the biscuits was then measured in millimeters using a computerized Vernier caliper. To ensure accuracy, two readings were taken by rotating the biscuits at a 90° angle. This process was repeated, and the average diameter was recorded in millimeters (Mahloko et al. 2019).

#### 2.8.2 Thickness

Three biscuits were vertically stacked (hereafter referred to as T), and their height was measured in millimeters (mm) using a Vernier caliper. This procedure was repeated three times to enhance accuracy, and the measurements were recorded in mm. The triplicate measurements were employed to compute the average value (Ambrose and Lekshman 2016).

#### 2.8.3 Spread Ratio

The spread ratio was calculated by dividing the diameter by thickness (Chinma and Gernah 2007).

(5) Spread ratio=DT

where D = the diameter of the biscuit; T = thickness of the biscuit.

#### 2.8.4 Texture Characteristics

The biscuits' texture was evaluated using the TA‐XT Plus texture analyzer model 12260 (Stable Micro Systems, Godalming, UK). A probe with a diameter of 2 mm and a length of 3 cm was inserted into the biscuit sample. The analysis included a pretest speed of 1.0 mm/s, a test speed of 0.55 mm/s, and a posttest speed of 10 mm/s. The texture analyzer was programed to measure the force exerted during the initial bite of the biscuit. The force‐time data were then analyzed to determine the hardness, which indicates the resistance to biting. This procedure was performed three times to ensure the reliability of the texture measurements (George 2020).

### 2.9 Color Properties

The color of biscuits was determined using a colorimeter (Lovibond LC 100 Spectrocolorimeter, England). The color was expressed as L* value (lightness) (+) and darkness (−); a* value (redness (+) and greenness (−)); b* value (yellowness (+) and blueness (−)); chroma; and hue (Thuwapanichayanan et al. 2011). The measurements were performed in triplicate, and the average values were reported as mean and standard deviation.

### 2.10 Microbial Quality

#### 2.10.1 *Escherichia coli* and Coliforms

A 2 g of biscuit samples was placed in 90 mL of distilled water and stirred to create a 10:1 dilution. Then, 1 mL of the dilution was pipetted into 9 mL of distilled water to make a 10:2 dilution. The same serial dilution process was followed to produce dilutions up to 10:5. The pour plate method was employed. For each dilution, 1 mL was pipetted onto a marked Petri dish. Approximately 20 mL of Violet Red Bile Agar (VBRA) was poured and mixed aseptically. After allowing the Petri dish to harden for a few minutes, it was incubated at 37°C for 24 h. The colony count was conducted after the 24‐h incubation period (Solaiman et al. 2020).

#### 2.10.2 Yeast and Mold

Ten grams of the sample was mixed with 90 mL of Buffered Peptone Water (BPW) and agitated for 2 min to ensure a uniform mixture. A series of dilutions were prepared by transferring a portion from one test tube to another. About 1 mL from each dilution was aseptically extracted using a sterile pipette and transferred onto triplicate plates. Subsequently, approximately 15 mL of potato dextrose agar (BBL, 211929) were poured onto the plates. The plates were then incubated for 5 days (Olee et al. 2022).

#### 2.10.3 Total Plate Count

Plate count agar (PCA) was prepared according to the manufacturer's guidelines. Subsequently, 1 ml of each respective serial dilution was carefully transferred onto the PCA using pipettes. The agar was then poured into Petri dishes and thoroughly mixed by rotating the dishes in both counterclockwise and clockwise directions. The Petri dishes were inverted and incubated at a temperature of 30°C for 24 h, as described by Nethathe et al. (2023). After the incubation period, the bacterial colonies were counted using a colony counter, and the results were expressed as colony‐forming units per gram (CFU/g) of the sample.

### 2.11 Statistical Analysis

The experiment was repeated three times, and all experimental data were collected in triplicate. Statistical analysis was performed using the Statistical Package for the Social Sciences (SPSS) version 26 by IBM Corp (2017). Various comparisons were made, including the functional properties, viscosity, and thermal properties of composite flour, as well as the proximate composition, antioxidant capacity, physical properties, color properties, and microbial quality of composite biscuits. One‐way analysis of variance (ANOVA) was used for these comparisons, and significant differences between treatment groups were assessed using Duncan's multiple range tests at a significance level of *p* < 0.05.

## 3 Results and Discussion

### 3.1 Functional Properties of Composite Flours

The functional properties of composite flours (Table 2) encompass various attributes that are crucial for its application in food production. Blending different flours to create a composite flour yields a product with distinct properties that impact aspects such as dough handling, texture, and sensory appeal. These properties include WAC, OAC, BD, dispersibility, and SC. They play a pivotal role in determining the suitability of composite flour for different food products. The exploration of functional properties not only fosters innovation in food formulation but also contributes to the development of diverse and nutritious food options for consumers. Regarding BD, the flour samples exhibited a range of 0.66–0.750 g/mL. There were no significant differences observed between control and flour samples added with 8% MPM and 2% OPFs, 16% MPM and 4% OPF, and 32% MPM and 8% OPFs (B, C, and E samples). On the other hand, sample D (24% MPM and 6% OPFs) had a lower BD than that of the control sample. Particle size and initial moisture content influence the BD of flour. Nonetheless, the particle sizes of the control and composite flour samples in this study were similar; therefore, variations in BD might be due to differences in initial moisture content. The low BD of sample D is advantageous for the preparation of weaning foods. This result is not in line with a report of Chandra et al. (2015) who reported an increase in the BD of composite flours with the decrease in percentages of WF.

**TABLE 2:** Functional properties of MPM–OP composite flours.

Sample	BD (g/mL)	WHC (g/g)	OAC (g/g)	SC (%)	Foaming capacity (%)	Dispersibility (%)
A	0.74 ± 0.12^a^	0.10 ± 0.06^c^	1.53 ± 0.12^d^	1.38 ± 0.01^a^	3.61 ± 0.08^a^	74.33 ± 0.52^a^
B	0.70 ± 0.07^ab^	0.10 ± 0.17^c^	1.60 ± 0.05^c^	1.31 ± 0.05^b^	3.57 ± 0.10^b^	66.00 ± 0.00^b^
C	0.70 ± 0.05^ab^	0.78 ± 0.25^b^	1.78 ± 0.12^ab^	1.32 ± 0.01^b^	3.57 ± 0.13^b^	64.67 ± 0.15^c^
D	0.66 ± 0.04^b^	1.07 ± 0.06^a^	1.90 ± 0.26^a^	1.25 ± 0.01^c^	3.66 ± 0.06^a^	67.00 ± 0.00^b^
E	0.75 ± 0.05^a^	1.15 ± 0.09^a^	1.57 ± 0.02^c^	1.38 ± 0.02^a^	3.42 ± 0.23^c^	67.33 ± 0.54^b^

*Note:* Mean values (*n* = 3) with by the same superscript(s) significantly differ within the similar column at *p* < 0.05. A = 100% WF, B = 90% WF + 8% MPM + 2% OPF, C = 80% WF + 16% MPM + 4% OPF, D = 70% WF + 24% MPM + 6% OPF, E = 60% WF + 32% MPM + 8% OPF.

Abbreviations: MPM, malted pearl millet; OAC, oil absorption capacity; OPF, orange powder flour; WF, wheat flour; WHC, water holding capacity.

There was a significant increase in WAC of the flour samples with the increase in percentages of MPM and OPFs with values varying from 0.10 to 1.15 g/g. The increase in WAC of composite flours might be due to high fiber content of MPM and OPFs. Nonetheless, the higher amount of hydroxyl groups in the fiber structure of both flours, which results in more interactions with water through hydrogen bonding, might also have contributed to the increase in WAC of composite flours (Rosell, Rojas, and De Barber 2001). The high capacity of composite flours to absorb water would be suitable for bakery products since they might reduce staling by preventing loss of moisture (Okpala, Okoli, and Udensi 2013).

The OAC of the flour is a critical functional property that influences its usage in food preparation and processing. This property refers to the ability of flour to absorb and retain oil or fats during mixing and cooking processes. The flour samples demonstrated a significant increase (*p* < 0.05) in OAC with the increase in the percentages of MPM and OPFs, with values ranging from 1.53 to 1.90 g/g. The increase in OAC of composite flour might be due to variations in the availability of nonpolar side chains of MPM and OP which might have bound the hydrocarbon side chains of oil in the flours (Mahloko et al. 2019). The results of this study show that incorporating MPM and OPFs could potentially improve the OAC of composite flours, thereby improving the texture, mouth feel, and flavor of biscuits (Godswill 2019). Flours with high OAC can be used in meat or bread products where fat absorption is desired. The obtained results for the composite flours are consistent with the reported results of 1.51–1.57 g/g for multigrain flours made from barley, sorghum, maize, oats, and wheat (Kumar et al. 2016). The results are also in line with the OAC of composite flours made from amaranth, rice, and soybean (Twinomuhwezi, Awuchi, and Rachael 2020). These flours promote structural interactions in foods, enhancing palatability, extending shelf life, and retaining flavors.

The SC of composite flour is a critical functional characteristic that determines its behavior when exposed to moisture. This property refers to the ability of composite flour to absorb water and expand, resulting in a viscous, gel‐like consistency. The SC is significant in various culinary processes, such as thickening, binding, and stabilizing food formulations. The SC of the flour significantly decreased with the increasing levels of MPM and OPFs with values ranging from 1.3 to 1.25 g/g. Nevertheless, no significant difference was observed between the SC of control and sample E (32% MPM + 8% OPFs). The variations in the SC of the flour might be due to the type of raw materials used and the processing method used. The SC is a consequence of noncovalent bonding between starch granule molecules and is influenced by variables that affect the amylose and amylopectin ratios (Hu et al. 2023). The obtained results were lower compared to those of the mixed flour of amaranth, rice, and soybean (Twinomuhwezi, Awuchi, and Rachael 2020).

The foaming capacity of composite flour is an important functional property that affects its suitability for various culinary applications. This property refers to how well composite flour can create stable foams when whipped or aerated. The foaming capacity of the composite flour samples ranged from 3.42% to 3.66% (samples A–E). The addition of MPM and OPFs increased the foaming capacity of flour samples. The high foaming capacity of composite flours demonstrate that they could form greater air bubbles surrounded by thinner a less flexible protein film (Chandra et al. 2015). Nonetheless, the foaming capacity is determined by the proteins' interfacial coating, which helps maintain air foams and prevent coalescence from occurring too quickly (Stochero, de Moraes, and de Oliveira 2020).

The results obtained were lower than those reported for composite flour made from pearl millet, buckwheat, amaranth, and unripe banana flour (Rustagi, Khan, and Jain 2022).

The dispersibility of composite flour is a crucial functional characteristic that affects its behavior when mixed with liquids. This property refers to how well the particles of composite flour can evenly disperse and hydrate in a liquid medium, resulting in a smooth and homogeneous mixture. The dispersibility of flour samples ranged from 64.67% to 74.33%. There was a significant decrease (*p* < 0.05) in dispersibility when MPM and OPFs were added. The lower dispersibility of the composite flour could be attributed to the higher percentages of MPM and OPFs, which may have reduced the impact of protein and, consequently, reduced dispersibility. This finding aligns with the results obtained by Melese and Keyata (2022). The dispersibility property of flour determines its ability to separate from water molecules and demonstrates its hydrophobic contact. Greater dispersibility as in control sample enhances the emulsifying and foaming capabilities of proteins, as observed in the production of bread, macaroni, and biscuits (Kaushik et al. 2021).

### 3.2 Viscosity of Hot and Cold Paste of Composite Flour

The cold paste showed different viscosities, ranging from 12.00 to 19.33 cP (Table 3). Adding MPM and OPFs resulted in a decrease in cold paste viscosity of flour samples. This decrease could be attributed to the presence of fiber in the MPM and OPFs, which enhances water absorption and subsequently reduces the viscosity of the paste. Fiber acts as a natural thickening agent and can affect the rheological properties of the paste. Furthermore, the addition of MPM and OPFs might have caused alterations in the starch content and structure of the WF. MPM and OP consist of distinct types of starch with varying properties that can influence the overall viscosity of the cold paste. Modifications in the composition and structure of the starch can impact the flow behavior and texture of the paste.

**TABLE 3:** Viscosity (hot and cold pastes) of composite flour.

Sample	Hot paste viscosity (cP)	Cold paste viscosity (CP)
A	74.33 ± 0.52^a^	19.33 ± 0.53^a^
B	66.00 ± 0.00^b^	18.33 ± 0.53^b^
C	66.67 ± 0.15^b^	13.00 ± 0.00^c^
D	67.00 ± 0.00^b^	12.33 ± 0.00^d^
E	67.33 ± 0.76^b^	12.00 ± 0.00^d^

*Note:* Mean values (*n* = 3) with by the same superscript(s) significantly differ within the similar column at *p* < 0.05. A = 100% WF, B = 90% WF + 8% MPM + 2% OPF, C = 80% WF + 16% MPM + 4% OPF, D = 70% WF + 24% MPM + 6% OPF, E = 60% WF + 32% MPM + 8% OPF.

Abbreviations: MPM, malted pearl millet; OPF, orange powder flour; WF, wheat flour.

The hot paste viscosity of the composite flour ranged from 66.00 to 74.33 cP. The inclusion of MPM and OPFs resulted in a reduction in the viscosity of the hot paste of the flour. This reduction might probably be due to the lower starch content in MPM and OPFs than in WF. Starch contributes significantly to the viscosity of hot pastes, and the lower starch content in the composite flour reduced the viscosity of hot pastes. Additionally, MPM and OP are rich in fiber, which has water‐binding properties.

The ability of the fiber to absorb water reduced the amount of free water and thus contributed to the reduced viscosity of the hot paste of composite flours. Furthermore, the particle size and distribution of flours used in the composite mixture influenced the viscosity of the hot paste. Finer particles and a more uniform distribution resulted in reduced viscosity due to increased water absorption and reduced starch gelatinization (Zhou et al. 2021). Lower viscosity is a reliable indicator of the acceptability of a weaning meal mixture for newborns, as stated in Olaleye, Oresanya, and Okwara (2020).

#### 3.2.1 Thermal Properties of Composite Flours

The thermal properties of composite flour (Table 4), including onset temperature, peak temperature, end temperature, and enthalpy, are crucial for comprehending its behavior during heating processes like baking or cooking. These properties offer insights into the flour's heat sensitivity, gelatinization behavior, and starch properties. The incorporation of MPM and OPFs increased the onset temperature of the flour with values ranging from 74.19°C to 89.35°C. This increase in starting temperature could be attributed to the possible change in starch gelatinization behavior caused by the presence of MPM and OPFs (Mashau et al. 2020). Additionally, the water content of the composite flour mixture can influence its thermal behavior. Adding MPM and OPF can result in a lower water content than WF alone, leading to an increase in the onset temperature. Water acts as a heat sink and affects the overall thermal properties of the mixture. Furthermore, the high fiber content in MPM and OPFs might create a more structurally complex network within the mixture, influencing heat transfer and thermal behavior.

**TABLE 4:** Thermal properties of composite flours.

Flour	Onset temperature	Peak temperature	End temperature	Enthalpy
	T_O_ (°C)	T_P_ (°C)	T_E_ (°C)	∆H (J/g)
A	74.19 ± 0.12^b^	79.30 ± 0.27^b^	83.51 ± 0.90^d^	4.80 ± 0.72^a^
B	84.09 ± 0.29^a^	91.09 ± 0.26^a^	95.97 ± 0.89^bc^	4.42 ± 0.31^a^
C	86.37 ± 0.69^a^	93.37 ± 0.18^a^	97.05 ± 0.88^b^	3.38 ± 0.56^b^
D	87.63 ± 0.55^a^	94.25 ± 0.32^a^	100.62 ± 0.68^a^	3.26 ± 0.35^b^
E	89.38 ± 0.21^a^	94.96 ± 0.30^a^	100.76 ± 0.89^a^	2.39 ± 0.55^c^

*Note:* Mean ± standard deviation. Values with the different superscript letters in the same column are significantly different at *p* < 0.05: T_O_ = onset temperature, T_O_ = peak temperature, T_E_ = end temperature ∆H enthalpy. A = 100% WF, Sample B = 90:8:2 (90%WF + 8% MPM + 2% OPF), Sample C = 80:16:4 (80% WF + 16% MPM + 4% OPF), Sample D = 70:24:6 (70% WF + 24% MPM + 6% OPF), Sample E = 60:32:8 (60% WF + 32% MPM + 8% OPF).

Abbreviations: MPM, malted pearl millet; OPF, orange powder flour; WF, wheat flour.

Regarding the peak melting temperature, Sample E (32% MPM + 8% OPFs) recorded the highest value at 94.96°C, while Sample A (control) recorded the lowest value at 79.30°C. There was a significant difference in peak melting temperature between the samples at *p* < 0.05. Adding MPM and OPF increased the peak melting temperature of the composite flour. This increase could be attributed to starch gelatinization, as MPM and WF contain gelatinous starch when heated. The presence of MPM and OPFs could have altered the gelatinization behavior of starch leading to changes in peak melting temperature. Additionally, changes in the water content due to the addition of MPM and OPFs could also influence melting behavior (Ma et al. 2022).

Considering the end temperature, Sample E (32% MPM + 8% OPFs) recorded the highest value at 100.76°C, while Sample A (control) recorded the lowest value at 83.51°C. All samples showed significant differences at *p* < 0.05. The addition of MPM and OPF increased the final finishing temperature of the composite flour. The temperature shift can be influenced by amylose and protein concentration, amylopectin branch chain distribution, and lipid complexes in the flour (Hellemans et al. 2020). High endothermic gelatinization (above 80°C) was observed in both the composite and WF, leading to increased competition for water in the system and an increase in the temperature of endothermic starch change (Mir, Gul, and Riar 2015).

The enthalpy values (ΔH) ranged from 2.39 to 4.80 (J/g), with a corresponding increase in MPM and OPFs. Significant differences at *p* < 0.05 were observed between Samples A–E. A decrease in enthalpy could depend on the specific proportions, processing methods, and interactions between ingredients in the composite flour (Donmez et al. 2021). The ∆H represents the energy required to break the intermolecular hydrogen bonds of the starch granules (Lv et al. 2022). This implies that changing the chemical composition of flour from organized to disordered requires little energy.

### 3.3 Proximate Composition of Composite Biscuits

Table 5 shows the proximate composition of biscuits made with a combination of MPM and OPFs. The moisture content of the biscuits ranged from 3.43% to 4.93%. Sample E (32% MPM + 8% OPFs) had the highest value, while Sample A (control) had the lowest value. Adding MPM and OPFs increased the moisture content of the biscuits. However, all samples remained below the maximum desired moisture content of 5% for biscuits (Mamat, Hardan, and Hill 2010). The moisture content falls within the range of 2.24%–3.32%, as reported by Mamat, Hardan, and Hill (2010). Keeping the moisture levels below this threshold is important to prevent damage from insects, bacteria, and mold during storage. The higher moisture content in Sample E could be attributed to its higher proportion of MPM flour, which might have enhanced water absorption due to the malting process. This improved WAC likely contributed to its higher moisture content compared to other samples (Onwurafor et al. 2022).

**TABLE 5:** Proximate composition of composite biscuits.

Sample	Moisture content (%)	Crude ash (%)	Crude fat (%)	Crude protein (%)	Carbohydrates (%)	Crude fiber (%)	Energy value (kcal)
A	3.43 ± 0.26^c^	4.50 ± 0.51^b^	9.20 ± 0.01^a^	11.70 ± 0.12^c^	65.46 ± 0.36^a^	11.44 ± 0.96^d^	393.80 ± 0.17^a^
B	3.88 ± 0.29^c^	5.13 ± 0.02^a^	8.28 ± 0.02^b^	11.90 ± 0.02^c^	63.77 ± 0.39^c^	13.00 ± 0.52^c^	385.17 ± 0.13^b^
C	4.00 ± 0.43^b^	5.19 ± 0.01^a^	7.49 ± 0.02^c^	12.03 ± 0.63^b^	64.85 ± 0.29^b^	15.98 ± 0.04^ab^	375.30 ± 0.26^c^
D	4.16 ± 0.51^b^	5.29 ± 0.20^a^	7.95 ± 0.06^c^	13.27 ± 0.01^a^	62.77 ± 0.43^cd^	15.06 ± 0.36^b^	362.58 ± 0.58^d^
E	4.93 ± 0.11^a^	5.54 ± 0.02^a^	7.35 ± 0.01^cc^	13.41 ± 0.01^a^	59.75 ± 0.55^e^	16.24 ± 0.74^a^	360.28 ± 0.81^e^

*Note:* Mean values (*n* = 3) with the same superscript(s) significantly differ within the similar column at *p* < 0.05. A = 100% WF, B = 90% WF + 8% MPM + 2% OPF, C = 80% WF + 16% MPM + 4% OPF, D = 70% WF + 24% MPM + 6% OPF, E = 60% WF + 32% MPM + 8% OPF.

Abbreviations: MPM, malted pearl millet; OPF, orange powder flour; WF, wheat flour.

The crude ash content of the biscuits ranged from 4.50% to 5.40%. The crude ash content increased as the proportion of MPM and OPFs increased in the biscuit formulation. This rise in ash content could be attributed to the higher mineral content present in the MPM and OP blends (Sobowale, Kewuyemi, and Olayanju 2021). OP is rich in potassium, calcium, magnesium, and phosphorus, while finger millet is rich in calcium and potassium (Rani, Sangwan, and Malik 2020; Mutshinyani, Mashau, and Jideani 2020). These findings provide insight into the nutritional attributes of composite flour and its potential contribution to the mineral content of the final product.

The crude fat content of biscuits refers to the total amount of lipid or fat present in the product before any specific extraction or purification process. It includes both saturated and unsaturated fats, which contribute to the texture, flavor, and nutritional profile of the biscuit. Analyzing the crude fat content provides insight into the energy density and dietary fat intake associated with consuming biscuits, which aids in nutritional assessment and formulating balanced diets. The crude fat content of the biscuits ranged from 7.35% to 9.20%. Sample A (control) had the highest value, while Sample E (32% MPM + 8% OPFs) had the lowest value. The inclusion of MPM and OPFs led to a reduction in the fat content of the flours. The decrease in the fat content in MPM could be attributed to the migration of lipids to the seed's rootlets during the malting process. On the other hand, the lower fat content in OPF could result from its inherently low fat content (Nwosu, Nweze, and Onwuchekwa 2022). The results obtained are lower than the 2.9% crude fat content reported by Alam et al. (2014). The MPM–OP blend offers healthier alternatives for consumers seeking lower‐fat food options. These findings highlight the positive impact of using MPM and OPF in creating nutritious and well‐balanced food products.

The crude protein content of biscuits is an essential aspect of their nutritional composition because it provides vital amino acids necessary for various physiological functions in the human body. The crude protein range in the composite biscuits varied from 11.70% to 13.41%. The inclusion of MPM and OPF blends increased protein content, with the control sample having the lowest value and Sample E having the highest value. It is important to note that OP typically has a low protein content, and the observed increase in protein levels could be attributed to the incorporation of MPM in the flour. The increased crude protein in the composite biscuits could be attributed to the malting process. During malting, leaching losses and protein translocation might have contributed to the overall increase in protein levels in biscuits (Hingade et al. 2019). Additionally, hydrolytic enzymes activated during malting might modify proteins, increasing the content of short peptides (Rani and Bhardwaj 2021). These biochemical transformations during malting likely contributed to the enhanced protein content observed in the biscuits enriched with MPM and OPFs. The obtained results for the biscuits fall within the range of 9.46%–20.45% reported by Makinde and Taibat (2018) for biscuits made from wheat, corn, almond, and coconut.

The crude fiber content of biscuits refers to the amount of indigestible plant material present in the product, including cellulose and lignin, which contribute to dietary fiber. The observed crude fiber content ranged from 11.44% to 16.24%. Sample E (32% MPM + 8% OPFs) had the highest dietary fiber value, while Sample A (control) had the lowest fiber content. The inclusion of mixtures of MPM and OP led to an increase in the crude fiber content of the samples, suggesting that these blends might be responsible for the observed increase. Generally, OP is rich in dietary fiber such as cellulose, hemicellulose, lignin, and pectin (Angel Siles López, Li, and Thompson 2010). The increase in crude fiber content in biscuits could be attributed to the conversion of insoluble fiber to soluble fiber during the malting process of pearl millet (Yang et al. 2021). The increased fiber intake from MPM and OPF is associated with various health benefits, including reduced risks of heart disease, diabetes, obesity, and certain types of cancers (Popoola‐Akinola, Raji, and Olawoye 2022). These findings exceed the 2.9% reported by Alam et al. (2014) for biscuits made from Holy Basil and *Moringa oleifera*. The crude fiber content of biscuits is highly relevant due to its impact on human health and the nutritional value of the product. Derived from plant‐based sources, crude fiber plays a crucial role in promoting digestive health, regulating blood sugar levels, managing weight, and reducing the risk of chronic diseases such as heart disease and diabetes.

The carbohydrates found in the composite biscuits serve as the main energy source and play a critical role in determining the texture and flavor of the biscuits. The carbohydrate content of the biscuits ranged from 59.46% to 65.58%. The inclusion of MPM and OP blends in the biscuits decreased the overall carbohydrate content of the biscuits. This decrease in carbohydrate levels was likely due to the integration of MPM and OPF in the formulation. The decrease in the carbohydrate content could be attributed to the enzymatic breakdown of starch into lower molecular weight sugars, such as maltose, glucose, and fructose (Onwurafor et al. 2020). This enzymatic conversion of starch into simpler sugars leads to an overall decrease in the carbohydrate content. The findings obtained for the composite biscuits were similar to the reported carbohydrate content of 78.71% for composite biscuits made from wheat, corn, almond, and coconut (Makinde and Taibat 2018).

The energy content of composite biscuits represents the total caloric value derived from their carbohydrate, fat, and protein content. The energy content of the biscuits ranged from 360.28 to 393.80 kcal. Among the samples of composite biscuits, Sample A (control) had the highest energy value, while Sample E (32% MPM + 8% OPFs) had the lowest energy value. The inclusion of MPM and OPF blends resulted in a reduction in the energy value of the biscuits. This difference in the energy content could be attributed to the relatively low‐calorie value of OP present in the biscuit (Elgindy 2020). These findings are consistent with the reported range of energy content for composite biscuits made from wheat, cassava, and soybean, which falls between 487.08 and 515.43 kcal (Okpalanma, Ishiwu, and Chukwu 2020). It is well‐established that the energy content of food is directly influenced by its protein, fat, and carbohydrate content (Shadieva et al. 2020).

### 3.4 Physical Properties of Composite Biscuits

The physical properties of composite biscuits (Table 6), such as diameter, thickness, distribution ratio, and hardness, are crucial in determining their quality, texture, and appeal to consumers. The diameter of the composite biscuits ranges from 4.02 to 4.60 mm, with no significant differences observed among biscuit samples. It is worth noting that the addition of MPM and OPFs did not affect the diameter of the biscuits.

**TABLE 6:** Physical properties of MPM–OPF composite biscuits.

Sample	Diameter (mm)	Thickness (mm)	Spread ratio (cm)	Hardness (N)
A	4.02 ± 0.21^ab^	9.00 ± 0.03^c^	4.64 ± 0.13^b^	7.53 ± 0.04^b^
B	4.24 ± 0.25^a^	9.67 ± 0.30^b^	4.29 ± 0.74^d^	7.59 ± 0.50^b^
C	4.26 ± 0.29^a^	10.00 ± 1.00^a^	4.20 ± 0.54^d^	8.00 ± 0.37^a^
D	4.33 ± 0.40^a^	9.00 ± 0.40^c^	4.98 ± 0.27^a^	8.00 ± 0.43^a^
E	4.60 ± 0.59^a^	10.00 ± 1.00^a^	4.50 ± 0.46^c^	8.75 ± 0.76^a^

*Note:* Mean values (*n* = 3) with the same superscript(s) significantly differ within the similar column at *p* < 0.05. A = 100% WF, B = 90% WF + 8% MPM + 2% OPF, C = 80% WF + 16% MPM + 4% OPF, D = 70% WF + 24% MPM + 6% OPF, E = 60% WF + 32% MPM + 8% OPF.

Abbreviations: MPM, malted pearl millet; OPF, orange powder flour; WF, wheat flour.

The thickness of the composite biscuits ranged from 9.00 to 10.00 mm. It is worth noting that Samples C (16% MPM + 4% OPFs) and D (24% MPM + 6% OP) flours had significantly greater thickness than the others, while Samples A (control) and E (32% MPM + 8% OPFs) had the thinnest biscuits, measuring 9.00 mm. The inclusion of MPM and OPF in the biscuit recipe might have contributed to these variations. The presence of gluten, a protein responsible for dough elasticity, could potentially reduce the thickness of the biscuits (Xu et al. 2020). These findings are consistent with previous study on unripe banana biscuits (Mashau, Rambau, and Kgatla 2022). The presence of alternative flours without gluten properties, such as MPM and OPF, might have weakened the formation of the gluten network and resulted in differences in biscuit thickness. It is important to remember that biscuit thickness can affect the sensory attributes and overall quality of the product. The observed variations in biscuit thickness among the composite biscuits highlight the importance of optimizing the formulation and processing parameters to achieve the desired product characteristics.

The spread ratio of the biscuits ranged from 4.20 to 4.98 cm. Sample D (24% MPM + 6% OPFs) had the highest expansion ratio, indicating that the biscuit expanded more during baking. Increase in spread ratio of this biscuit sample might be attributed to poor association between the network of protein and carbohydrates (Mahloko et al. 2019). Moreover, spread ratio is related to the texture, bite, and overall mouth feel of biscuits (Bose and Shams‐Ud‐Din 2010). However, the spread ratio of other composite biscuits was lower than that of control.

The decreased spread ration in composite biscuits might be attributed to competition for the available water by ingredients that can absorb water during baking. Moreover, composite flours, due to the different WAC of their ingredients, form aggregates, thereby increasing the number of hydrophilic sites that can interact with water. These findings are consistent with previous study on biscuits made from unripe bananas and WFs (Mashau, Rambau, and Kgatla 2022).

The hardness of the composite biscuits ranged from 7.53 N to 8.75 N. These variations in hardness were found to be statistically significant at *p* < 0.05, indicating that the inclusion of MPM and OPF had a substantial impact on the hardness of the biscuits. This increase in hardness could be attributed to the combination of wheat protein and pearl millet flour protein, which leads to a compression effect during biscuit formation and, ultimately, an improvement in hardness properties. The presence of fiber in the composite biscuits might have also contributed to their hardness (Kulthe, Thorat, and Lande 2017; Mashau, Rambau, and Kgatla 2022). Hardness is a critical structural characteristic of biscuits that significantly influences sensory perception and overall product quality.

### 3.5 Bioactive Compounds and Antioxidant Activity of Biscuits

The bioactive compounds and the antioxidant activity of biscuits were evaluated (Table 7). The TPC of the biscuits ranged from 1.40 to 3.63 mg GAE/100 g. Among the samples, Sample D (24% MPM + 6% OPFs) exhibited the highest TPC value, indicating a significant concentration of phenolic compounds with potential antioxidant activity. The increase in TPC of composite biscuits might be attributed to that OP contains a wide variety of value‐added compounds, including organic acids, flavonoids, and polyphenols. Moreover, malting might have activated enzymes that break down and alter the cell wall of the pearl millet grain, thereby enhancing the phenolic content of the resultant flour used for biscuits making (Arya 2022; Murungweni, Ramashia, and Mashau 2024). Conversely, Sample A (control sample) recorded the lowest TPC value. This low TPC value might be due to that the composition of the phenolic compounds varies among the flours and some of them may decompose and volatilize during baking (Chandra et al. 2015). This finding aligns closely with previous research on biscuits fortified with *Citrus bergamia*, which reported a value of 5.84 mg GAE/100 g (Laganà et al. 2022). Phenolics are well‐known antioxidant molecules associated with cancer prevention and act through various mechanisms to combat infections (George 2020).

**TABLE 7:** Antioxidant and bioactive compounds of composite biscuits.

Sample	TPC (mg GAE/100 g)	TFC (mg QUE/100 g)	Vitamin C (mg/g)	FRAP (mg GAE/g)
A	1.40 ± 1.79^b^	2.91 ± 0.52^d^	0.79 ± 0.07^b^	1.78 ± 0.36^d^
B	3.16 ± 1.04^a^	3.97 ± 0.60^c^	1.01 ± 0.06^a^	3.23 ± 0.98^c^
C	3.57 ± 1.46^a^	2.20 ± 0.46^e^	1.01 ± 0.02^a^	3.46 ± 0.39^c^
D	3.63 ± 1.09^a^	5.22 ± 0.41^b^	1.01 ± 0.01^a^	5.13 ± 0.01^b^
E	3.56 ± 1.45^a^	6.79 ± 0.17^a^	1.01 ± 0.01^a^	8.64 ± 0.21^a^

*Note:* Mean values (*n* = 3) with the same superscript(s) significantly differ within the similar column at *p* < 0.05. A = 100% WF, B = 90% WF + 8% MPM + 2% OPF, C = 80% WF + 16% MPM + 4% OPF, D = 70% WF + 24% MPM + 6% OPF, E = 60% WF + 32% MPM + 8% OPF.

Abbreviations: FRAP, Ferric reducing antioxidant power; MPM, malted pearl millet; OPF, orange powder flour; TFC, total flavonoid content; TPC, total phenolic content; WF, wheat flour.

The TFC values of biscuits ranged from 2.20 to 6.79 mg QUE/100 g with Sample C (16% MPM + 4% OPFs) having the lowest value and Sample E (32% MPM + 8% OPFs) having the highest value. The decrease in TFC of Sample E might be due to enzymatic activity during the malting process of pearl millet. On the other hand, the increase in TFC of composite biscuits might be due to incorporation of OP since it is rich in flavonoids. Consumption of these composite biscuits may offer potential health benefits due to the interaction of flavonoids with various biological systems. Flavonoids are well known for their antioxidant properties, ability to scavenge‐free radicals, protection against oxidative cell damage, and strong anticancer effects (Sarkar et al. 2022). The obtained results are comparable with those of biscuits incorporated with *Parinari curatellifolia* flour (Ramashia, Mamadisa, and Mashau 2021).

The ascorbic acid in composite biscuits enhances its nutritional and functional qualities. As a potent antioxidant, ascorbic acid, or vitamin C, it safeguards against oxidative damage and supports various bodily functions, including collagen synthesis, immune health, and wound healing. The ascorbic acid content of the biscuits in this study ranged between 0.79 and 1.01 mg/g. The incorporation of MPM and OPFs significantly increased the ascorbic acid of the biscuits. This was anticipated since OP is a rich source of vitamin C. Sir Elkhatim, Elagib, and Hassan (2018) reported that 100 g of the whole peel of oranges contains110.4 mg of ascorbic acid. Ascorbic acid is known for its effective quenching of singlet oxygen and other radicals. Additionally, ascorbic acid is recognized for promoting the absorption of inorganic iron and inhibiting the production of nitrosamines in the stomach (Anyiam et al. 2023). Results of this study align with previously reported findings on yellow corn and cowpea biscuits and wheat–cocoyam composite flour (Irondi et al. 2021; Makanjuola and Adebowale 2020).

The FRAP antioxidant capacity of the biscuits significantly increased with the increasing percentages of MPM and OPFs with values ranging from 1.77 to 8.64 mg GAE/g. The increase of FRAP might be due to increase in TPC and TFC of composite biscuits. Moreover, heat during baking disrupts the cell wall, allowing more antioxidant compounds to be released, resulting in increased antioxidant activity (Mudau, Mashau, and Ramashia 2022). The chemicals present in the biscuits seem to function more efficiently through the hydrogen atom transfer mechanism. These findings align with a study conducted by Jan et al. (2015). The inclusion of buckwheat flour at 20% and 40% in WF led to higher FRAP values for the composite cookies. Furthermore, the FRAP values surpassed those observed in cookies made from *Parinari curatellifolia* flour (Ramashia, Mamadisa, and Mashau 2021).

### 3.6 Color Attributes of Composite Biscuits

Table 8 shows the color profile of biscuits. The color of food plays a crucial role in the consumer's initial perception of its quality. Even before the product is introduced to the market, its appearance and color acceptance are vital factors that must be considered (Leon et al. 2006). The L* value of the composite biscuit significantly decreased ranging from 31.90 to 55.96 to 31.90, as shown in Table 8. Sample E (32% MPM + 8% OPFs) had the lowest L* value, while Sample A (control) had the highest value. The increase in dark color (low L* value) could be associated with the addition of seed coat material, which tends to darken when heated (Krishnan, Rayaguru, and Nayak 2020). Moreover, this could also be attributed to the pectin content in OPF, as discussed by Mahmoud, Abou‐Arab, and Abu‐Salem (2015).

**TABLE 8:** Color properties of composite biscuits.

Samples	L*	a*	b*	H°	Chroma	∆E
A	55.96 ± 0.89^a^	15.20 ± 0.80^a^	36.85 ± 0.24^a^	52.05 ± 0.51^a^	67.56 ± 0.72^a^	—
B	55.16 ± 0.81^a^	13.32 ± 0.63^b^	32.58 ± 3071^b^	45.88 ± 0.49^b^	64.21 ± 0.69^b^	50.50 ± 0.02^d^
C	42.12 ± 0.13^b^	12.48 ± 0.18^c^	23.88 ± 0.08^d^	36.36 ± 0.26^d^	61.70 ± 0.76^d^	59.00 ± 0.66^c^
D	43.20 ± 0.50^b^	13.41 ± 0.12^b^	26.69 ± 0.69^c^	40.10 ± 0.17^c^	63.29 ± 0.76^c^	65.53 ± 0.59^a^
E	31.90 ± 0.63^c^	10.82 ± 0.99^d^	19.59 ± 0.26^e^	30.42 ± 0.19^e^	53.66 ± 0.36^e^	60.36 ± 0.19^b^

*Note:* Mean values (*n* = 3) with the same superscript(s) significantly differ within the similar column at *p* < 0.05. A = 100% WF, B = 90% WF + 8% MPM + 2%OPF, C = 80% WF + 16% MPM + 4% OPF, D = 70% WF + 24% MPM + 6% OPF, E = 60% WF + 32% MPM + 8% OPF.

Abbreviations: MPM, malted pearl millet; OPF, orange powder flour; WF, wheat flour.

With regard to a* value, Sample A (control) had the highest a* value at 15.20, while Sample E (32% MPM + 8% OPFs) had the lowest a* value at 10.82. Increasing the proportion of MPM and OPF decreased the a* content of the composite biscuits. This decrease in a* could be attributed to various factors, including the Maillard browning reaction, natural pigments, baking temperature and time, and the overall color composition. There was a significant decrease in b* value with the increasing levels of MPM and OPFs with values ranging from 36.85 to 19.59. The browning effect of MPM might have contributed to the decreased in b* value of composite biscuits. Thermal degradation of carotenes and xanthophyll in OPFs might also have contributed to the decrease in b* values. Nevertheless, the decreased b* value of composite biscuits might be attributed to both MPM and OPFs since they are rich source of polyphenols which contribute to the dark color (Mala, Piayura, and Itthivadhanapong 2024). On the other hand, WF contains yellow pigments (xanthophyll) and might have contributed to its high b* value (Mala, Piayura, and Itthivadhanapong 2024).

The addition of MPM and OPFs led to a decrease in hue angle with values varying from 52.05 to 30.42. Hue represents the qualitative aspect of the color of the biscuits. Hue angle is a qualitative characteristic of color, traditionally defined as red, green, or blue. Hue is an important aspect of color perception and plays a role in the acceptance of the color of the biscuits for individuals with normal color vision. Sample A (control) exhibited the highest chroma value (67.56) among all samples. Meanwhile, the minimum chroma value for composite biscuits was 53.66 (Sample E, 32% MPM + 8% OPF). It is evident that there was a significant difference between all samples at a *p* < 0.05 level. The chroma value of the biscuits decreased as the MPM and OPF increased. The decrease in chroma and hue of the composite biscuits might be attributed to the addition of MPM and a modification in color pigments during malting caused by the oxidation of phenolic acid (Taylor and Duodu 2015). In the study, the authors noticed that all composite biscuit samples exhibited chroma values, and higher MPM and OPF values resulted in lower chroma values.

The color difference (ΔE*) of the biscuits samples significantly increased with values varying from 50.50 to 65.53. These observed color changes in biscuit samples could be attributed to the browning effect caused by Maillard reaction compounds that form during the drying of the peels. For the human eye, ΔE values of composite biscuits higher than 3 indicate an obvious color difference. These findings are consistent with a study conducted by Mashau, Rambau, and Kgatla (2022), who also reported decreased L*, a*, and b* values in wheat biscuits prepared with unripe bananas.

### 3.7 Microbial Quality of Composite Biscuits

The microbial quality of composite biscuits, including total plate count, coliforms, *Escherichia coli*, yeast, and mold, is presented in Table 9. All the samples showed a significant difference at *p* < 0.05. The total plate count is a commonly used method for evaluating the hygienic quality of food. It involves isolating microorganisms, both pathogenic and nonpathogenic, that thrive in aerobic and temperate conditions (20°C–45°C) (Hanum, Kurniawati, and Normaliska 2018). For the composite biscuits, the total plate count ranged from 4.304 to 8.33 log_10_ CFU/g. Significantly, there were notable variations in total plate count among all samples, with a significance level of *p* < 0.05. It is worth mentioning that an increase in the proportions of MPM and OPFs resulted in a decrease in the total plate count content in the composite biscuits. This reduction in total plate count might be attributed to certain ingredients in the composite biscuit formulation, such as OP, which possesses natural antimicrobial properties. OPF contains essential oils and compounds like limonene, which are known for their antimicrobial effects. These properties inhibit the growth of microorganisms and reduce the overall bacterial count (Xu et al. 2020).

**TABLE 9:** Microbial quality of composite biscuits (log_10_ cfu/g).

Sample	Total plate count	Coliforms	*Escherichia coli*	Yeast	Mold
A	8.34 ± 0.52^a^	8.23 ± 0.31^a^	1.50 ± 0.71^d^	8.67 ± 0.06^a^	6.00 ± 0.61^a^
B	4.5 0 ± 0.20^d^	5.67 ± 0.04^b^	2.67 ± 0.09^c^	7.00 ± 0.61^b^	ND
C	4.30 ± 0.50^d^	4.00 ± 0.20^c^	5.27 ± 0.52^b^	5.00 ± 0.36^c^	5.67 ± 0.73^b^
D	6.03 ± 0.89^b^	ND	8.17 ± 0.10^a^	4.67 ± 0.31^d^	2.67 ± 0.08^c^
E	5.17 ± 0.25^c^	ND	0.33 ± 0.58^e^	0.67 ± 0.16^e^	ND

*Note:* Mean values (*n* = 3) with the same superscript(s) significantly differ within the similar column at *p* < 0.05. A = 100% WF, B = 90% WF + 8% MPM + 2% OPF, C = 80% WF + 16% MPM + 4% OPF, D = 70% WF + 24% MPM + 6% OPF, E = 60% WF + 32% MPM + 8% OPF.

Abbreviations: MPM, malted pearl millet; ND, not detected; OPF, orange powder flour; TPC, total plate count; WF, wheat flour.

Considering coliform counts, the biscuits exhibited coliform counts ranging from 0.0 to 8.23 log10 CFU/g. Samples D and E showed no presence of coliform bacteria, while Sample A exhibited the highest coliform count at 8.23 log_10_ CFU/g. The lowest count was observed in sample C at 4.00 log_10_ CFU/g. Sample A demonstrated a significantly higher count than the other samples in terms of the mean coliform count. The incorporation of MPM and OPFs resulted in a reduction in the coliform count of the composite biscuits. These findings are consistent with previous studies on biscuits made from buckwheat and corn—African yam and cassava bark, respectively. It is important to note that coliform counts serve as indicators of fecal contamination, even though their presence does not always indicate the presence of disease (Mritunjay and Kumar 2017; Some et al. 2021).

The levels of *E. coli* in the biscuits ranged from 0.33 to 8.17 log_10_ CFU/g. Sample D (24% MPM + 6% OPFs) had the highest value, while Sample E (32% MPM + 8% OPFs) had the lowest value. The inclusion of MPM and OPFs in the biscuits impacted the presence of *E. coli*. These findings demonstrate higher *E. coli* counts compared to those reported by Owheruo et al. (2023). Moreover, the *E. coli* counts in the biscuits exceeded the acceptable limit of < 2 log10 CFU/g, as indicated by Ayamah, Sylverken, and Ofori (2021). It is worth mentioning that the presence of *E. coli* in biscuits does not necessarily indicate the presence of pathogenic microorganisms. Nonetheless, it suggests the potential existence of fecal contamination (Odonkor and Mahami 2020).

The addition of MPM and OPFs led to a decrease in yeast content in the final biscuit product. This decrease could be attributed to the presence of OP in the formulation, which have inherent antimicrobial properties (Giri et al. 2023). These antimicrobial properties have the potential to inhibit yeast growth and activity, resulting in a decrease in yeast functionality. OPF contains compounds, like essential oils, known to have antimicrobial effects against yeast. Additionally, the composition of the flours used in the biscuit formulation, including wheat, MPM, and OPFs, can also affect yeast activity. Different flours have varying levels of enzymes, nutrients, and gluten, which can influence yeast fermentation.

Regarding mold, Samples A (8% MPM + 2%OPFs), C (16% MPM + 4% OPFs), and D (24% MPM + 6% OPFs) had mold, while Samples B (8% MPM + 2% OPFs) and E (32% MPM + 8% OPFs) showed no mold. Sample A had the highest proportion of mold at 6.00 log_10_ CFU/g, while Sample D had the lowest at 2.67 log_10_ CFU/g. The use of MPM and OPFs in the composite biscuits resulted in a decrease in mold infestation. It is important to note that molds can produce mycotoxins, which can cause food spoilage and foodborne illnesses when present in high concentrations (Shankar et al. 2021). Therefore, reducing mold in food is crucial for ensuring food safety and quality. The findings of this study suggest that substituting WF with blends of MPM and OPFs can effectively reduce the microbial content in composite biscuits. This reduction in microbial counts, including bacteria, yeast, and molds, in the substituted WF biscuits might be due to phytochemicals in MPM and OP. These phytochemicals possess inhibitory properties against harmful microorganisms in flour (Adugna, Neme, and Abera 2022).

## 4 Conclusions

The study investigated the functional properties, viscosity, and thermal properties of composite flour. Additionally, the study examined the proximate composition, physical properties, antioxidant properties, color attributes, and microbial safety of biscuits made from MPM flour and OPFs. Based on the results, the composite flour showed increased WAC, dispersibility, and foaming capacity, while SC, oil holding capacity, and BD decreased with the inclusion of MPM and OPFs. Furthermore, the hot and cold viscosity of the composite flour decreased. Regarding the thermal properties, the composite flour indicated an increase in onset, peak, and end temperatures, while enthalpy decreased upon the incorporation of MPM and OPFs. Notable variations were observed in the proximate composition of the biscuits. Specifically, there was an increase in moisture content, crude protein, and crude fiber. At the same time, a decrease was observed in crude fat, carbohydrates, and energy values with the addition of MPM and OPFs. In terms of the physical properties of the composite biscuits, there was an increase in diameter, thickness, and hardness, while the spread ratio decreased with the inclusion of MPM flour and OPFs. Furthermore, the addition of MPM and OPFs resulted in an augmentation of the antioxidant properties of the biscuits, as evidenced by an increase in TPC, total flavonoid content, and ferric‐reducing antioxidant power. Regarding the color attributes of the composite biscuits, there was a decrease in L*, a*, b*, hue, and ΔE due to the inclusion of MPM and OPFs. Lastly, the microbial quality of the composite biscuits showed a reduction in total counts, coliform, *Escherichia coli*, and yeast with an increase in MPM and OPFs. These research findings hold valuable implications for food manufacturers as they can enhance product quality and functionality, thereby reducing reliance on wheat.

## Author Contributions

**Shonisani Eugenia Ramashia:** funding acquisition (lead), investigation (equal), methodology (equal), project administration (lead), supervision (lead), validation (equal), visualization (equal), writing – review and editing (equal). **Matimu Delicate Ntsanwisi:** conceptualization (equal), data curation (equal), formal analysis (equal), methodology (equal), writing – original draft (lead). **Oluwatoyin Oladayo Onipe:** investigation (equal), visualization (equal), writing – review and editing (equal). **Mpho Edward Mashau:** investigation (equal), validation (equal), visualization (equal), writing – review and editing (equal). **Gbeminiyi Olamiti:** investigation (equal), methodology (equal), validation (equal), visualization (equal), writing – review and editing (equal).

## Conflicts of Interest

The authors declare no conflicts of interest.

## Data Availability

There is no primary data related to this manuscript.
